# Nitrogen-doped Carbon Derived from ZIF-8 as a High-performance Metal-free Catalyst for Acetylene Hydrochlorination

**DOI:** 10.1038/srep39789

**Published:** 2017-01-04

**Authors:** Songlin Chao, Fang Zou, Fanfan Wan, Xiaobin Dong, Yanlin Wang, Yuxuan Wang, Qingxin Guan, Guichang Wang, Wei Li

**Affiliations:** 1 College of Chemistry, Key Laboratory of Advanced Energy Materials Chemistry (Ministry of Education), Nankai University, Tianjin, 300071, China; 2 Collaborative Innovation Center of Chemical Science and Engineering, Tianjin, 300071, China

## Abstract

Acetylene hydrochlorination is a major industrial technology for manufacturing vinyl chloride monomer in regions with abundant coal resources; however, it is plagued by the use of mercury(II) chloride catalyst. The development of a nonmercury catalyst has been extensively explored. Herein, we report a N-doped carbon catalyst derived from ZIF-8 with both high activity and quite good stability. The acetylene conversion reached 92% and decreased slightly during a 200 h test at 220 °C and atmospheric pressure. Experimental studies and theoretical calculations indicate that C atoms adjacent to the pyridinic N are the active sites, and coke deposition covering pyridinic N is the main reason for catalyst deactivation. The performance of those N-doped carbons makes it possible for practical applications with further effort. Furthermore, the result also provides guidance for designing metal-free catalysts for similar reactions.

Vinyl chloride monomer (VCM) is an important commodity chemical that is used to synthesize poly(vinyl chloride) (PVC). In developing countries with abundant coal reserves, acetylene hydrochlorination is the preferred manufacturing process, using HgCl_2_ supported on activated carbon as the catalyst. However, the catalyst is environmentally toxic with relatively short lifetimes because the HgCl_2_ is apt to sublime during reaction^1^. Recently, about 140 countries accepted “The Minamata Convention on Mercury” and promised to reduce and eventually abolish the use of mercury by 2020^2^. Therefore, the development of nonmercury catalysts is the top priority for the industry in the process of acetylene hydrochlorination.

Gold catalysts have been proven to be the best replacement of mercury for this reaction^3^,^4^,^5^ and extensive work has been carried out to decrease the gold content and improve the stability of the gold catalyst^6^,^7^,^8^,^9^. Hutchings, the pioneer of gold catalyst research, has been close to achieving the commercialization of gold catalysts in collaboration with Johnson Matthey^10^. However, considering the limitation of the price and reserves of noble metals, development of further alternatives remains highly desirable.

The development of catalysts made with more abundant elements and even metal-free catalysts is the optimal choice for the reaction. The unique catalytic properties of carbon materials have been extensively demonstrated for a variety of reactions, and it has been proved that N-doped carbon materials are active for acetylene hydrochlorination^1^,^2^. Bao *et al*. reported that a N-doped carbon derived from silicon carbide worked as a catalyst for the reaction, the acetylene conversion reaching 80% at 200 °C. Theoretical simulations led to the conclusion that carbon atoms bonded with pyrrolic N atoms are the active sites^2^. Wei *et al*. reported that N-doped carbon nanotubes are active for acetylene hydrochlorination and the quaternary N is the biggest contributing N-containing form^11^. Dai *et al*. synthesized different N-doped carbons through adopting N-doping treatments on activated carbon, and the results demonstrated that pyrrolic N is the most important nitrogen species^12^. In addition, they also pointed out that pyridinic N at the edge of a N-doped graphene oxide had a specific and dominant HCl adsorption capacity that is positive for the reaction of acetylene hydrochlorination^13^. Although all of this outstanding work has been explored, the role of different N species (pyrrolic N, pyridinic N, and quaternary N) in active sites is still under debate^11^,^13^,^14^, and the practical applications of N-doped carbon materials are limited by relatively low conversion, poor stability, or low production capacity^2^,^11^,^13^.

Herein, we report a N-doped carbon catalyst for acetylene hydrochlorination with both high activity and quite good stability (the acetylene conversion reached 92% and decreased slightly during a 200 h test at 220 °C, atmospheric pressure, and 1.4 mL g^−1^ min^−1^ of C_2_H_2_, corresponding to a space velocity of 30 h^−1^ based on the catalyst volume). Experimental studies and theoretical calculations indicate that C atoms adjacent to the pyridinic N are the active sites, and coke deposition covering pyridinic N is the main reason for catalyst deactivation.

## Results

**Figure 1:**
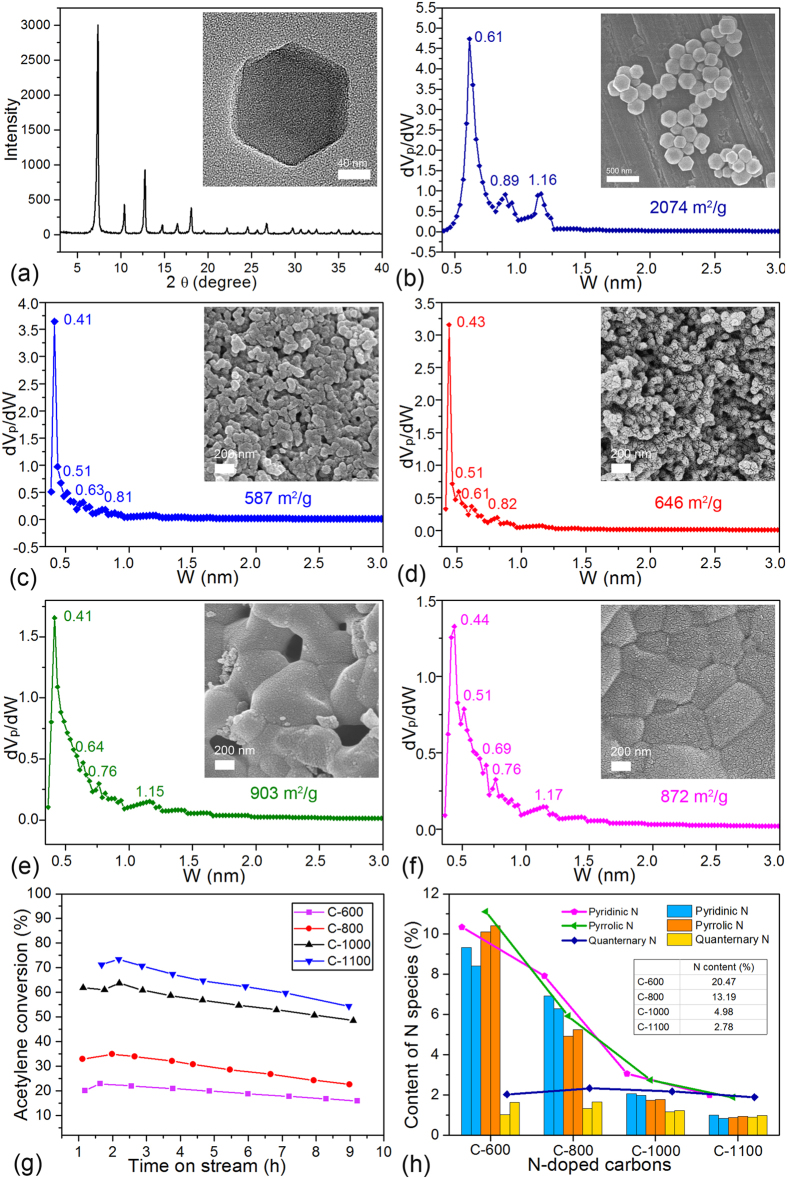
(**a**) XRD pattern and TEM image of ZIF-8. (**b**–**f**) Pore information and SEM images of ZIF-8, C-600, C-800, C-1000 and C-1100. (**g**) Acetylene conversion by N-doped carbon. Reaction conditions: temperature = 220 °C, a space velocity of C_2_H_2_ = 4.7 mL g^−1^ min^−1^, feed volume ratio between HCl and C_2_H_2_ = 1.2. Due to the different bulk density, the corresponding C_2_H_2_ gas hourly space velocity (GHSV) of C-600, C-800, C-1000 and C-1100 is: 129 h^−1^, 112 h^−1^, 105 h^−1^ and 87 h^−1^, respectively. (**h**) N content analysis base on XPS. (1) N content in the table comes from fresh N-doped carbons. (2) The broken line shows content change trend of the specific N species with the temperature increases. (3) Histograms exhibit content of specific N specie in different N-doped carbons, and for the adjacent two same color pillars, the left represent N specie content of fresh carbons meanwhile the right represent that of used carbons.

For N-doped carbon materials used as catalysts, large specific surface and high dispersion of N species are crucial factors affecting catalyst activity and stability. Based on this consideration, the ZIF-8 is a good candidate to synthesize N-doped carbons directly upon carbonization because of the multiple advantages of ZIF-8—large surface area, regular structure, and a high N content^15^,^16^,^17^,^18^. Initially, ZIF-8 was synthesized in methanol at room temperature (for details see the Supplementary Information). The synthesis route was selected because the synthesis reaction could be promoted by molecular interactions between the reagents and the solvent with hydrogen-bond donation ability, which could improve textural properties and produce a smaller particle size of ZIF-8^19^,^20^. According to the results of X-ray diffraction (XRD), scanning electron microscope (SEM), transmission electron microscope (TEM), and nitrogen adsorption/desorption measurements shown in Fig. 1a and b, the prepared ZIF-8 has pure phases, ordered crystalline shapes with a particle size of about 200 to 300 nm, and abundant pore structures with the surface area up to 2074 m^2^ g^−1^.

### Effect of calcination temperature on materials’ morphology and catalytic activity

To investigate the influence of calcining temperature on carbon materials, ZIF-8 was carbonized at different temperatures (600, 800, 1000, and 1100 °C) under flowing N_2_ to obtain N-doped carbons with different N-species content and texture properties^21^. The choice of temperature is based on the result of thermogravimetric analysis (TGA) (See Supplementary Fig. S1) that the mass loss of ZIF-8 mainly occurs after heating to 600 °C. It is important to emphasize that the ZIF-8 was kept for at least 1 h at 300 °C for better properties of the carbon materials (See detailed process in the Supplementary Information). The pore texture information and SEM images of obtained N-doped carbons (C-600, C-800, C-1000, and C-1100) are shown in Fig. 1c–f, respectively. Compared with ZIF-8, the obtained carbons exhibit a significant decrease in surface area, and the average pore size shifts from 0.61 to 0.40 nm or so. This is because the framework of ZIF-8 has collapsed at the carbonization temperatures, and the porosity of N-doped carbons comes from cracks in the framework and the evaporation of Zn metal during pyrolysis^16^. In addition, washing with hydrochloric acid after the carbonization process removed residual Zn in those carbons and provided additional pore structure at the same time.

The set of N-doped carbons (0.3 g) was tested for acetylene hydrochlorination at 220 °C and an acetylene space velocity of 4.6 mL g^−1^ min^−1^ (For details see the Supplementary Information). The results are shown in Fig. 1g. It should be mentioned that catalytic selectivity of all the catalysts tested in this study is virtually 100%. The initial acetylene conversion of the N-doped carbons has a positive correlation with the carbonization temperature, and the conversion of C-600, C-800, C-1000, and C-1100 is ca. 23%, 35%, 65%, and 75%, respectively. Based on the data shown in Fig. 1c–f, different catalytic activity mainly comes from the difference in surface area and pore structure. Furthermore, it has been proved that doping N species play the main role in forming the active sites for acetylene hydrochlorination^1^,^2^. To investigate the effects of N species on catalytic activity, X-ray photoelectron spectroscopy (XPS) analyses of fresh and used N-doped carbon catalysts were carried out. According to the bonding state of N atoms, the N 1 s spectra were deconvolved into three individual component peaks: pyridinic N (398.3–399.8 eV), pyrrolic N (400.1–400.5 eV), and quaternary N (401–402 eV)^2^,^11^,^17^,^22^. The statistics of the change of different N-doped carbons is summarized in Fig. 1h, and see the detailed curve fitting in Supplementary Fig. S2. According to the results of XPS, total N content of the set of N-doped carbons is 20.47%, 13.19%, 4.98%, and 2.78%, respectively. The decrease of N content with the increase of temperature is because nitrogen in the ZIF-8 is carried off in the airflow when the framework is destroyed at high temperature. According to the histogram, the percentage of pyridinic N decreased, whereas the pyrrolic and quaternary N increased after reaction. Based on the report that coke formation covering the active sites was the main reason for deactivation^13^, it seems that for the N-doped carbon catalysts, the deactivation was caused by coke deposition on pyridinic N, which is responsible for creation of the active site. To sum up, the N-doped carbons obtained at elevated temperature have low pyridinic N content but high reaction conversion may be because carbons calcined under elevated temperatures have a more microcrystalline structure and higher specific surface area, which could provide more defects and effective N species for acetylene hydrochlorination (For more discussion see the Supplementary Information Figs S3 and S4).

### Long-term stability test of the improved catalyst and study of the deactivation mechanism

To improve the catalytic activity further, ZIF-8 was calcined under an NH_3_ atmosphere because NH_3_ could expand the micropore size-distributions and increase the surface defects^23^. In addition, to make the material synthesis more efficient and convenient, ZIF-8 was prepared using a solid-state synthesis, in which ZnO was mixed with ligands and heated directly to yield ZIF-8^16^,^20^. Although there are some differences in the crystal size and crystallinity of ZIF-8 synthesized via different routes (See Supplementary Fig. S5), the N-doped carbons calcined under N_2_ atmosphere show consistent catalytic activity. The ZIF-8 synthesized using the solid-state method was calcined at 1000 °C under N_2_ and NH_3_ atmospheres, and the obtained carbons (referred to as C-N_2_, C-NH_3_) were tested for long-term stability for acetylene hydrochlorination. C-N_2_ (1 g, ca. 2.6 mL) and C-NH_3_ (1 g, ca. 2.8 mL) were tested in a fixed-bed microreactor with the HCl at 1.7 mL min^−1^ and C_2_H_2_ at 1.4 mL min^−1^, corresponding to a space velocity of about 30 h^−1^.

**Figure 2:**
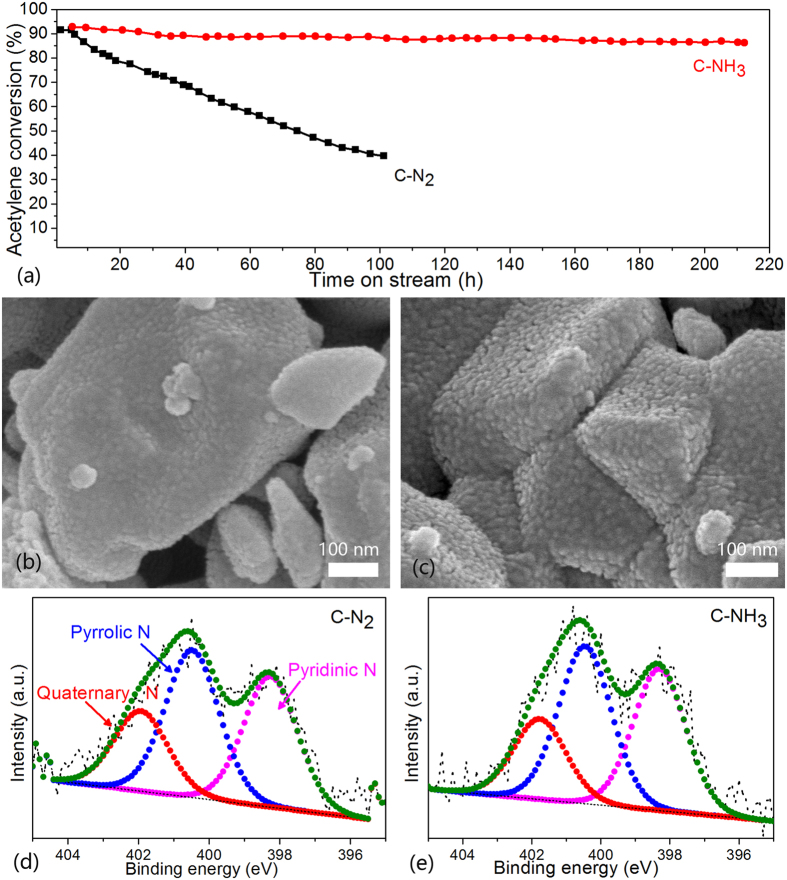
(**a**) Acetylene conversion by N-doped carbon. Reaction conditions: temperature = 220 °C, a space velocity of C_2_H_2_ = 1.4 mL g^−1^ min^−1^, feed volume ratio between HCl and C_2_H_2_ = 1.2. (**b**–**d**) SEM images of C-N_2_ and C-NH_3_. (**e**) and (f) N 1 s XPS of C-N_2_ and C-NH_3_.

Obviously, C-NH_3_ exhibits high acetylene conversion of about 92% and a rather good stability with the conversion only decreasing slightly during a 200 h test, whereas the C-N_2_ shows basically the same initial activity but poor stability, as demonstrated in Fig. 2a. For N-doped carbon catalysts, pore structure and the active N species are the main factors affecting catalyst activity. According to Fig. 2b and c, the C-NH_3_ has a larger specific surface area (1166.6 m^2^ g^−1^) than C-N_2_ (875.5 m^2^ g^−1^), which mainly comes from the greater micropore structure. From the SEM pictures, C-NH_3_ also reveals more abundant texture structure, which may be from the activation of ammonia^24^. On the other hand, C-NH_3_ and C-N_2_ have basically the same N content (6.28% for C-N_2_ and 4.59% for C-NH_3_ according to the results of XPS) and similar N-species proportion, as shown in Fig. 2d and e. In sum, C-NH_3_ has better catalytic stability than C-N_2_ as it has a larger surface area and similar N content. However, it is still not clear what the decisive factor for the difference in stability is.

The loss of active component is the main reason for catalyst deactivation in industrial use of mercury catalyst. However, the N-doped carbon catalyst obtained by calcination at elevated temperatures has a more stable structure compared with the supported mercury catalyst. The results of element analysis (6.35% for C-N_2_ and 4.03% for C-NH_3_, 5.89% for the used C-N_2_ and 3.85% for the used C-NH_3_) reveals that the N contents of C-N_2_ and C-NH_3_ have a slight change after the long-term test. And the reducing of N contents should mainly be caused by the carbon deposition increasing the quality of the samples rather than by the physical loss.

**Figure 3:**
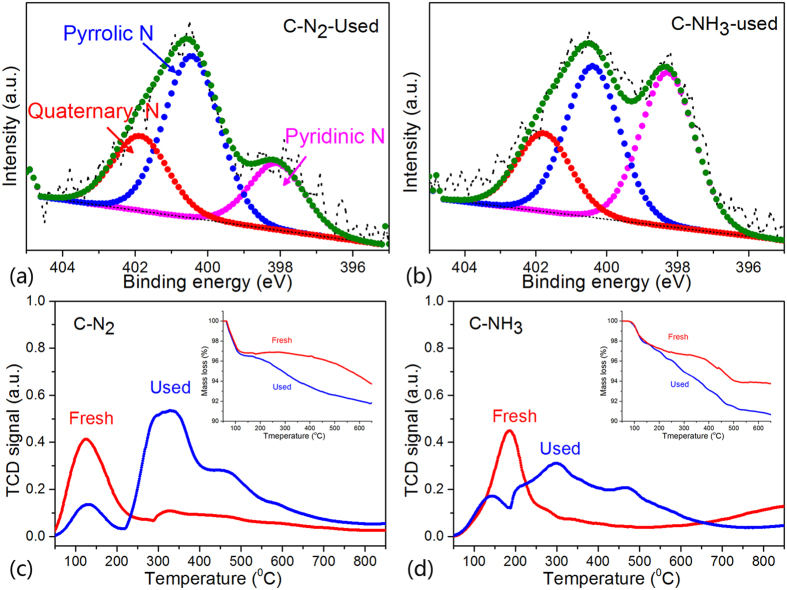
(**a**) and (**b**) N 1 s XPS of used C-N_2_ and C-NH_3_. (**c**) and (**d**) C_2_H_2_–TPD and (**b**) TG analysis of used C-N_2_ and C-NH_3_.

To understand the difference further, the C-N_2_ and C-NH_3_ were characterized with XPS, TGA, and temperature-programmed desorption (TPD) using C_2_H_2_ as the probe. It is worth noting that the proportion of pyridinic N in C-N_2_ reduced from 36.6% to 21.9% after a 100 h test; meanwhile, the pyridinic N ratio of C-NH_3_ basically remained unchanged after the 200 h test (Fig. 3a and b). It is consistent with the statistical result shown in Fig. 1h, which means that deactivation was caused by coke deposition on pyridinic N, which is responsible for creation of the active site. According to the C_2_H_2_ TPD of fresh C-N_2_ and C-NH_3_ (Fig. 3c and d), there is only one desorption peak below 200 °C, which represents the adsorption and activation of C_2_H_2_^25^. However, there is another peak between 200 and 500 °C for the catalysts used, and the peak area below 200 °C is lower than that of the fresh catalyst. The decrease of peak area below 200 °C represents the reduction of the ability to adsorb and activate C_2_H_2_, which may be caused by covering of the active sites. The peak between 200 and 500 °C should come from the decomposition of coke deposition combining with the results of TGA in Fig. 3c and d. Combined with the results of XPS, it might be concluded that coverage of the pyridinic N is the reason for catalyst deactivation.

Two points should be noted: the results of C_2_H_2_ TPD show that the peak between 200 and 500 °C of used catalyst could be convolved into multiple desorption peaks and the surface area is far larger than the area of the peak below 200 °C. This may be because the coke deposition is a polymer^10^, which would decompose giving more gaseous product than the adsorbed C_2_H_2_ under the TPD process. In addition, the nitrogen adsorption data that the surface area of C-N_2_ decreases from 875.5 to 264.5 m^2^ g^−1^ after catalytic reaction while the surface area of C-NH_3_ decreases from 1166.6 to 617.0 m^2^ g^−1^ also demonstrated that coke deposition is the main reason for catalyst deactivation. However, it is still unclear why the C-N_2_ and C-NH_3_ have a small difference in surface area and pyridinic N content, but huge difference in catalytic stability.

### Reaction mechanism studied by density functional theory (DFT)

**Figure 4:**
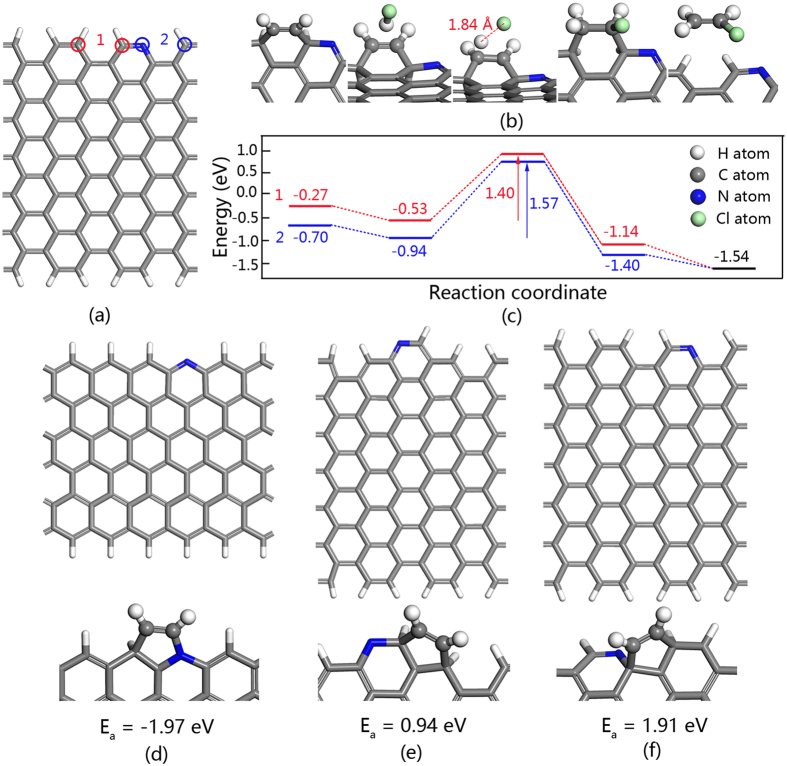
DFT calculations on the reaction mechanism. (**a**) Structure models with pyridinic N, with the blue representing N atoms, the grey representing C atoms, the white representing H atoms. Sites 1 denotes two neighbor C atoms; sites 2 stands for pyridinic N atom and neighbor C atom. (**b**) The possible catalytic pathway basing on sites 1. (**c**) Reaction energy profile on sites 1 and 2 from DFT calculations. (**d**–**f**) Other structure models of pyridinic N.

DFT calculations were carried out for further understanding of the difference in catalytic stability. Both theory and experiments indicate that carbon atoms bound to N are the active sites in N-doped carbon^1^,^2^,^11^,^22^. Thus, various structure models of pyridinic N have been inspected. Effective models and two possible catalytic pathways are shown in Fig. 4a,b and c. The first step in the reaction is most likely to be the addition of C_2_H_2_ to the potential active sites (Fig. 4a, sites 1 and 2) and the corresponding data are shown in Fig. 4c (−0.27 eV and −0.70 eV correspond to sites 1 and 2, respectively). Subsequently, HCl approaches the catalyst with dissociative adsorption energies of −0.53 eV and −0.94 eV. The reactants need to surmount an additional barrier (1.40 eV and 1.57 eV, respectively) to achieve the transition state structures, and then a CH_2_CHCl molecule is formed with adsorption energies of −1.14 eV for site 1 and −1.40 eV for site 2. Finally, the formed CH_2_CHCl escapes from the catalyst and the product is obtained. Comparing the different reaction processes, the pathway corresponding to site 1 with a barrier of 1.40 eV is the most favorable reaction route on N-doped carbon for acetylene hydrochlorination. For the structure model of pyridinic N shown in Fig. 4d, the energy of acetylene adsorption on zigzag edges of N-doped carbon is −1.97 eV. This is a very stable configuration, which will make vinyl chloride stripping difficult. Polymerization of C_2_H_2_ or VCM may be caused in this type of site. This may be the reason for coke deposition and catalyst deactivation. For structures shown in Fig. 4e and f, it is hard to adsorb acetylene with energies of 0.94 eV and 1.91 eV. Those may be some of the reasons why some N-doped carbons with high pyridinic N content show low reaction conversion.

## Discussion

Combining with the DFT calculations and the experimental data, it seems large enough surface area and effectively pyridinic N provide a similar catalytic activity for C-N_2_ and C-NH_3_. And the difference in pyridinic N structures make C-N_2_ easier to cause coke deposition than C-NH_3_, which is the reason caused the differences between the stability of those two N-doped carbon catalysts. However, there is no clear evidence to prove the hypothesis. It need more sophisticated model catalyst and higher precision of spectral characterization to distinguish the certain pyridinic N structures, and then determine the relationship between the certain pyridinic N structure and the catalytic activity. However, it a challenge to control the generation of certain N structures.

In summary, it has been demonstrated that the synthesis of N-doped carbon using ZIF-8 as the precursor is a convenient and feasible strategy for preparing highly efficient catalysts for acetylene hydrochlorination. The N-doped carbon derived from ZIF-8 calcined at 1000 °C exhibits high acetylene conversion of ca. 92% and outstanding stability during a 200 h test at 220 °C, and a space velocity of 30 h^−1^. The results of analysis on deactivation of the catalyst indicate that coverage of the pyridinic N is the main reason for catalyst deactivation. The DFT showed that only the specific pyridinic N structure is effective for creating active sites; meanwhile, other structures can produce coke deposits easily. In-depth studies of ZIF-derived carbon for practical application to acetylene hydrochlorination are in progress in our group.

## Additional Information

**How to cite this article**: Chao, S. *et al*. Nitrogen-doped Carbon Derived from ZIF-8 as a High-performance Metal-free Catalyst for Acetylene Hydrochlorination. *Sci. Rep.* **7**, 39789; doi: 10.1038/srep39789 (2017).

**Publisher's note:** Springer Nature remains neutral with regard to jurisdictional claims in published maps and institutional affiliations.

## Supplementary Material

Supplementary Information
