# Supplementary material for: Nitrogen-doped Carbon Derived from ZIF-8 as a High-performance Metal-free Catalyst for Acetylene Hydrochlorination
Source: Sci Rep. 2017 Jan 4;7:39789. doi: 10.1038/srep39789 (PMC5209682; doi:10.1038/srep39789)
Supplement: Supplementary Information [file srep39789-s1.pdf]

Supplementary Information

Nitroge-doped Carbon Derived from ZIF-8 as a High-performance Metal-free Catalyst for Acetylene Hydrochlorination

Songlin Chao,[a] Fang Zou,[a] Fanfan Wan,[a] Xiaobin Dong,[a] Yanlin Wang,[a] Yuxuan Wang,[a] Qingxin Guan,[a] Guichang Wang*[a] and Wei Li*[a][b]

Characterization data and discussion

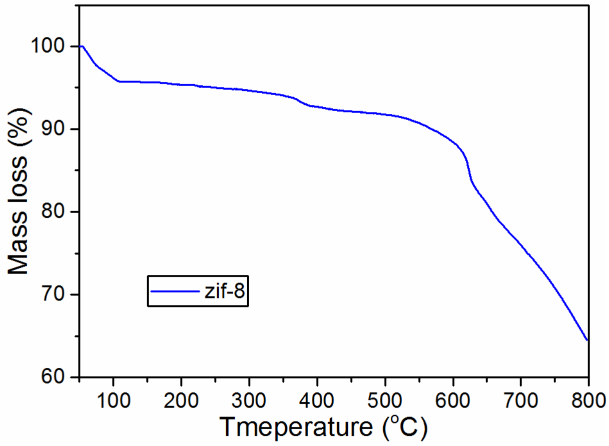

Fig. S1. TGA curves recorded in atmosphere N_2 of ZIF-8.

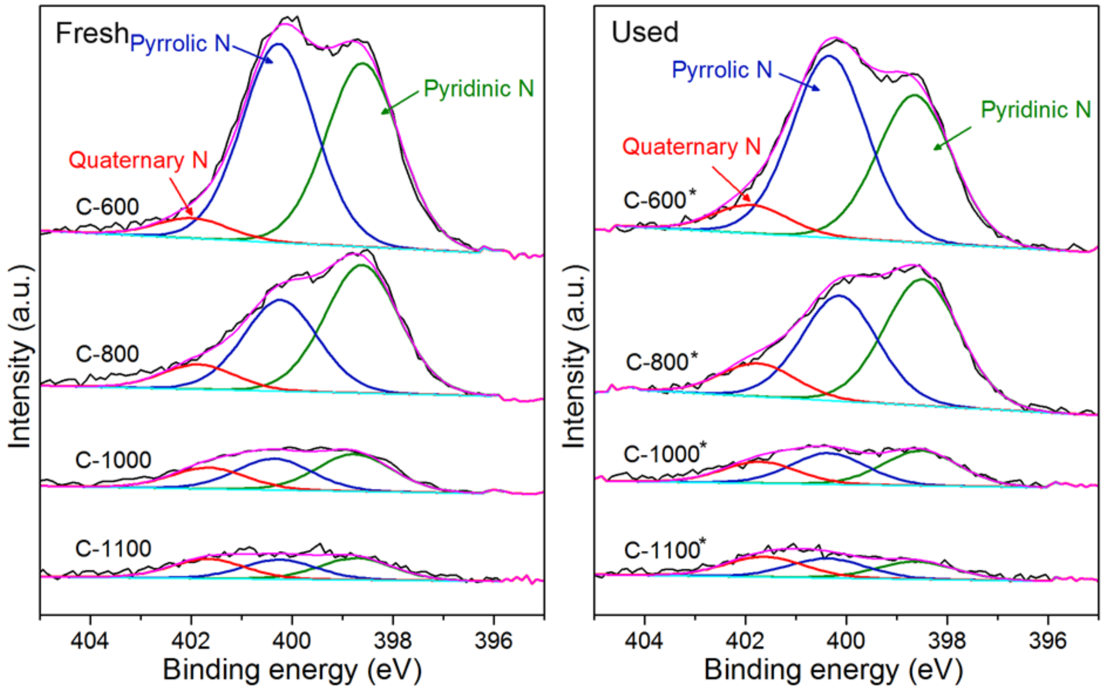

Fig. S2. XPS N 1s spectra of fresh and used N-doped carbons.

According to the results of XPS, the total N content of different N-doped carbons is 20.47%, 13.19%, 4.98% and 2.78%, respectively. There is a decrease downward trend of N con-tent, which is consistent with the results of elemental analysis that the N content of C-600, C-800, C-1000 and C-1100 is 27.4%, 18.4%, 7.5% and 3.52%, respectively.

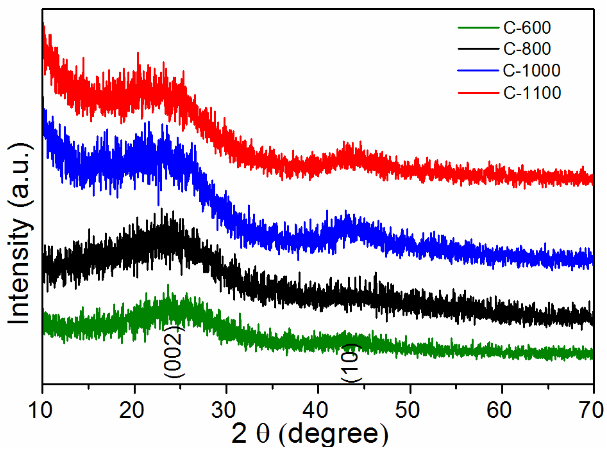

Fig. S3. XRD patterns of N-doped carbons.

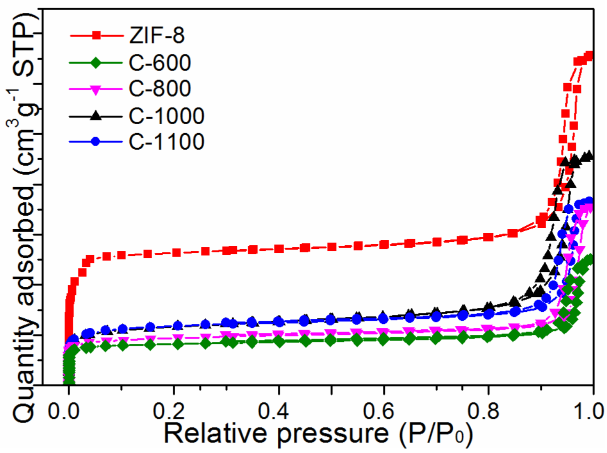

Fig. S4. Nitrogen adsorption–desorption isotherms of ZIF-8 and N-doped carbons.

The observed XRD curves show a broad peak at (002) and (10) positions, that could be analyzed to get the crystallite size and their distributions. The interlayer spacing d, and the crystallite size L_c, are obtained from the (002) band. The average layer diameter, L_a(10) is obtained from the (10) band.[14,15]

For the bands of (002), the peak intensity of C-1000 and C-1100 increased slightly compared to that of C-600 and C-800. It indicates that there is increase in crystallite size with the calcination temperature increasing. In addition, there is obvious peak at

(10) position in curves of C-1000 and C-1100. Combined with the bands of (002), it demonstrated that the interlayer spacing and average layer diameter increased with the temperature increasing, which represented the stacking pore increased in C-1000 and C-1100. The observations are consistent with the results in Fig. 1. From Fig. 1c, d, e and f, it is obvious that the proportion of pores with a size of around 0.4 nm decreased and the proportion of pores between 0.4 and 1.2 nm increased, with the increase of calcination temperature. Combined with the SEM images, it seems that ZIF-8 cannot be carbonized adequately below 800 °C. When being calcined at 1000 °C, this material was in a molten-like state and stacking pores appeared. This is the reason why the number of pores with sizes between 0.4 and 1.2 nm increased. However, the surface area of C-1100 is lower than that of C-1000. This may be caused by some micropore structure coming from the break of the framework being blocked by the molten-linked carbons.

The result of elemental analysis display that N content of C-600, C-800, C-1000 and C-1100 is 27.4%, 18.4%, 7.5% and 3.52%, respectively. The difference of the numerical value between elemental analysis and XPS is because XPS is a surface technique whereas elemental analysis is a bulk tech-nique using different working principle. Based on Fig 1f, in the perspective to understand the effect of N species on active site, two points should be mentioned: (1) according to the broken lines, the content of pyridinic N and pyrrolic N decreased dramatically with the increase of calcination temperature, however, the content of quaternary N basically remain unchanged. (2) According to the histogram, the percentage of pyridinic N reduced

whereas the pyrrolic and quaternary N increased after reaction evaluating.

On the basis of the two phenomena, there are two assump-tions were taken into considered: (1) the quaternary N plays the crucial role in acetylene hydrochlorination. The results of the N species vary with temperature are because that pyridinic and pyrrolic N can be thermally decomposed, mean-while quaternary N is the most thermally stable species and other species can reassembles into this structure. The different catalytic activity of the set of N-doped carbons with similar content of quaternary N is caused by different texture character (surface area, pore size distribution and bulk den-sity). (2) The pyridinic N plays the crucial role in acetylene hydrochlorination. When using metal catalysts in acetylene hydrochlorination, loss of the active metal or coke deposition occupying active site is the possible cause of catalyst deactivation. For the N-doped carbon catalysts, the deactivation was caused by coke deposition on pyridinic N, which is responsible for creation of active site. The results that the N-doped carbons obtained at elevated temperature with low pyridinic N content but high reaction conversion may because carbons calcined under elevated temperatures have more microcrystalline structure (Fig. S3 and S4) and higher specific surface area (Fig. 1). Those characters make the carbons have more effective pyridinic N in edge of crystal.

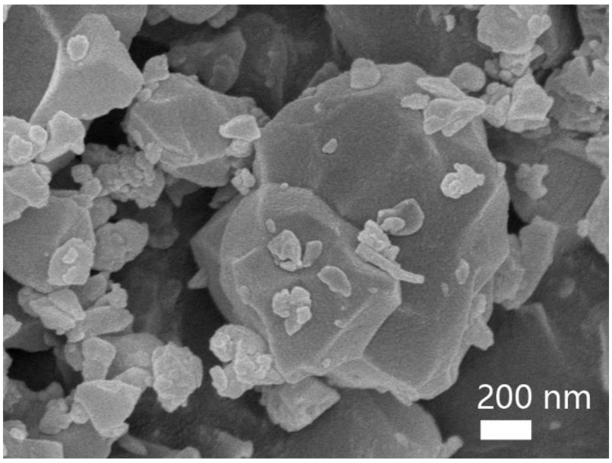

Fig. S5. SEM of ZIF-8 synthesized with the solid state.

Experimental section

Synthesis of ZIF-8 in a methanol system. Typically, a solution of $Zn(NO_3)_2 \cdot 6H_2O$ (7.350 g, 0.0247 mol) in 500 mL methanol was rapidly poured into a solution of 2-methylimidazole (16.2 g, 0.1973 mol) in 400 mL methanol under stirring with a magnetic bar. The resulting mixture was kept undisturbed for 3 h at ambient temperature[2][1], followed by high-speed centrifugation to separate the nanocrystals from the milky dispersion. The obtained nanocrystals were washed twice with fresh methanol and then dried at 80 °C for 12 h.

Synthesis of ZIF-8 with the solid state. Typically, imidazole (13.6 g, 0.2 mol) and ZnO (8.1g, 0.1 mol) were combined, grided, and sealed in an autoclave under N_2 atmosphere. The mixture was heated at 180 °C for 12 hours. The product was obtained as white powder. [3]

Preparation of N doped carbons. The ZIF-8 was placed in a quartz tube, under N_2 flowing at 50 mL min^{-1} for 1 h to exclude air at ambient temperature, then heated to 300 °C at the rate of 5 °C min^{-1} and kept for 1 h under flowing N_2. Subsequently, the material was carbonized under flowing N_2 at different temperatures (600, 800, 1000 and 1100 °C) for 5 h with a heating rate of 5 °C min^{-1}. After being cooled to ambient temperature in situ, the obtained materials were washed thoroughly in HCl (4 M) to remove the remnants of Zn. Then, the obtained N-doped carbons were dried at 130 °C for 10 h, and referred to as C-600, C-800, C-1000 and C-1100, respectively.

Catalytic activity test. The obtained N-doped carbons were directly used as the

catalysts for acetylene hydrochlorination and the catalysts were tested in a fixed-bed microreactor. In general, 0.3 g of catalysts were mixed with silica sand to expand the volume to 2 mL. Silica sand was also used above the catalysts, which could mix and preheat the reactants. Prior to the reaction, the carbon catalysts were pretreated in situ with HCl (1.7 mL min^{-1}) at 220 °C for 1 h. After that, HCl (1.7 mL min^{-1}) and C_2H_2 (1.4 mL min^{-1}) were fed to the heated reactor via calibrated mass flow controllers. A blank experiment was carried out using an empty reactor filled with silica sand under the same conditions, and the silica sand did not show any catalytic activity. The gas products were analyzed online using a gas chromatograph equipped with a thermal conductivity detector (TCD)

Characterization of N doped carbons. Brunauer–Emmett–Teller (BET) specific surface area data were obtained using nitrogen adsorption/desorption measurements at 77 K with a BELSORP-Max instrument, and the micropore size distribution analysis from the adsorption isotherm was calculated using the Horvath–Kawazoe (HK) method. The size and morphology of those materials were examined using a SEM (JSM-7500F, JEOL), or a transmission electron microscope (Tecnai G2 F20, FEI). Elemental analysis was performed with an elemental analyzer (EA, Vario EL CUBE, Elementar). Thermogravimetric analysis was conducted with a thermogravimetric analyzer derivative thermogravimetry analyzer (TG–DTA, S60, SETARAM). X-ray photoelectron spectroscopy (XPS) spectra were obtained using an Axis Ultra DLD spectrometer with a monochromatized Al Kα X-ray source (250 W). Temperature programmed desorption (TPD) analysis was carried out using a Micromeritics

Chemisorb 2750 instrument equipped with a TCD. [4]

DFT calculations. The periodic, self-consistent DFT calculations were performed using the Vienna Ab initio Simulation Package (VASP). [5-7] The exchange–correlation effects have been described within the generalized gradient approximation (GGA), using the Perdew, Burke and Ernzerhof (PBE) [8] functional. The electron–core interaction was described by the projector-augmented plane-wave (PAW) [9,10] method with a cutoff energy of 400 eV. The Brillouin zone was sampled with 3 × 1 × 1 Monkhorst–Pack [11] mesh k-points. The convergence test of energy and force were set to 1×10^{-4} eV and 0.035 eV/Å, respectively. The DFT-D3 [12] method was used to add van der Waals correction to the DFT calculations. The climbing-nudged elastic-band method (cNEB) [13] was employed to locate the transition state (TS), and a frequency analysis was carried out to confirm the TS. All of the ab initio molecular dynamics (AIMD) simulations were carried out using an NVE ensemble with a 1.0 fs time step and a 1 ps duration. The DFT calculation models were set in rectangular supercells. The vacuum thicknesses in the directions perpendicular and parallel to the ribbon plane were both set at 12 Å. The armchair edges are six benzene rings in width. AIMD was performed to investigate the stability of models at the reaction temperature.

1 Zhang, L. J. et al. Highly graphitized nitrogen-doped porous carbon nanopolyhedra derived from ZIF-8 nanocrystals as efficient electrocatalysts for oxygen reduction reactions. Nanoscale 6, 6590-6602, (2014).

2 Venna, S. R., Jasinski, J. B. & Carreon, M. A. Structural Evolution of Zeolitic Imidazolate Framework-8. J. Am. Chem. Soc. 132, 18030-18033, (2010).

3 Zhao, D. et al. Highly Efficient Non-Precious Metal Electrocatalysts Prepared from One-Pot Synthesized Zeolitic Imidazolate Frameworks. Adv. Mater. 26, 1093-1097, (2014).

4 Chao, S., Guan, Q. & Li, W. Study of the active site for acetylene hydrochlorination in AuCl3/C catalysts. J. Catal. 330, 273-279, (2015).

5 Kresse, G. & Hafner, J. \textit{Ab initio} molecular dynamics for liquid metals. Physical Review B 47, 558-561 (1993).

6 Kresse, G. & Furthmüller, J. Efficiency of ab-initio total energy calculations for metals and semiconductors using a plane-wave basis set. Computational Materials Science 6, 15-50, (1996).

7 Kresse, G. & Furthmüller, J. Efficient iterative schemes for \textit{ab initio} total-energy calculations using a plane-wave basis set. Physical Review B 54, 11169-11186 (1996).

8 Perdew, J. P., Burke, K. & Ernzerhof, M. Generalized Gradient Approximation Made Simple. Phys. Rev. Lett. 77, 3865-3868 (1996).

9 Kresse, G. & Joubert, D. From ultrasoft pseudopotentials to the projector augmented-wave method. Physical Review B 59, 1758-1775 (1999).

10 Monkhorst, H. J. & Pack, J. D. Special points for Brillouin-zone integrations. Physical Review B 13, 5188-5192 (1976).

11 Mills, G. & Jónsson, H. Quantum and thermal effects in ${\mathrm{H}}_{2}$ dissociative adsorption: Evaluation of free energy barriers in multidimensional quantum systems. Phys. Rev. Lett. 72, 1124-1127 (1994).

12 Grimme, S., Ehrlich, S. & Goerigk, L. Effect of the damping function in dispersion corrected density functional theory. J. Comput. Chem. 32, 1456-1465, (2011).

13 Mills, G., Jónsson, H. & Schenter, G. K. Reversible work transition state theory: application to dissociative adsorption of hydrogen. Surf. Sci. 324, 305-337, (1995).

14 Aso, H., Matsuoka, K., Sharma, A. & Tomita, A. Structural analysis of PVC and PFA carbons prepared at 500-1000 degrees C based on elemental composition, XRD, and HRTEM. Carbon 42, 2963-2973, (2004).

15 Sharma, A., Kyotani, T. & Tomita, A. Comparison of structural parameters of PF carbon from XRD and HRTEM techniques. Carbon 38, 1977-1984, (2000).
